# Healthy Diet, Polygenic Risk Score, and Upper Gastrointestinal Cancer Risk: A Prospective Study from UK Biobank

**DOI:** 10.3390/nu15061344

**Published:** 2023-03-10

**Authors:** Wenmin Liu, Tianpei Wang, Meng Zhu, Guangfu Jin

**Affiliations:** 1 Department of Epidemiology, School of Public Health, Nanjing Medical University, Nanjing 211166, China; 2 Jiangsu Key Lab of Cancer Biomarkers, Prevention and Treatment, Collaborative Innovation Center for Cancer Personalized Medicine and China International Cooperation Center for Environment and Human Health, Nanjing Medical University, Nanjing 211166, China; 3 Public Health Institute of Gusu School, The Affiliated Suzhou Hospital of Nanjing Medical University, Suzhou 215000, China

**Keywords:** UGI cancer, dietary pattern, polygenic risk score, prospective cohort, UK Biobank

## Abstract

Dietary and genetic factors are considered to be associated with UGI cancer risk. However, examinations of the effect of healthy diet on UGI cancer risk and the extent to which healthy diet modifies the impact of genetic susceptibility on UGI cancer remains limited. Associations were analyzed through Cox regression of the UK Biobank data (*n* = 415,589). Healthy diet, based on “healthy diet score,” was determined according to fruit, vegetables, grains, fish, and meat consumption. We compared adherence to healthy diet and the risk of UGI cancer. We also constructed a UGI polygenic risk score (UGI-PRS) to assess the combined effect of genetic risk and healthy diet. For the results high adherence to healthy diet reduced 24% UGI cancer risk (HR _high-quality diet_: 0.76 (0.62–0.93), *p* = 0.009). A combined effect of high genetic risk and unhealthy diet on UGI cancer risk was observed, with HR reaching 1.60 (1.20–2.13, *p* = 0.001). Among participants with high genetic risk, the absolute five-year incidence risk of UGI cancer was significantly reduced, from 0.16% to 0.10%, by having a healthy diet. In summary, healthy diet decreased UGI cancer risk, and individuals with high genetic risk can attenuate UGI cancer risk by adopting a healthy diet.

## 1. Introduction

Upper gastrointestinal (UGI) cancer, including esophageal cancer (ESC) and gastric cancer (GC), account for 1.7 million new cancer cases and 1.3 million deaths each year worldwide [1]. Previous studies have identified several common environmental risk factors for UGI cancer, including tobacco [2] and alcohol consumption [3], obesity [4], physical activity [5], and dietary factors [6]. Dietary components have received an increasing amount of attention as a potentially modifiable factor [7,8].

It was estimated that 5.1–5.9% of cancer cases each year worldwide can be attributed directly to poor diet [9]. As recently reported by the World Cancer Research Fund International/American Institute for Cancer Research, the role of individual dietary components on UGI cancer risk remains controversial and limited [10]. Rather than individual dietary components, people consume diverse foods together, and the resulting complex combination of dietary components is likely to have interactive or synergistic effects [11]. In this context, dietary pattern analysis has been recommended as an approach because it considers the complexity of overall diet and can potentially facilitate public health interventions [12]. In recent years, cancer prevention guidelines have shifted from reductionist or nutrition-centric approaches to more holistic dietary concepts characterized by dietary patterns. Holistic dietary concepts emphasize how food as a whole can prevent chronic disease, associating nutrients, foods or food groups with health rather than studying the role played by nutrient/food interactions in health [13,14,15]. Adherence to a dietary pattern can be assessed using a priori method, which is constructed on the basis of a predefined set of criteria (generally based on guidelines) to measure diet quality in a given population [16], which would be easier to make comparisons between different studies and populations. A meta-analysis of the association of GC risk with dietary patterns indicated that Western dietary patterns (generally considered unhealthy, characterized by an increased consumption of meat, high-fat dairy products, sweets, and starchy foods) were associated with a higher GC risk, while prudent dietary patterns (generally considered healthy, characterized by higher intake of vegetables and fruits) played a protective factor [17]. A case-control study suggested that adherence to a healthy dietary pattern represented by high loadings of vegetables and fruits was associated with a lower risk of GC [18]. However, there is no large-scale prospective cohort study that systematically investigates the association between dietary patterns and UGI cancer risk.

Accumulating evidence has shown that genetic factors have major roles in the development of UGI cancer [19,20]. Recent genome-wide association studies (GWAS) have identified dozens of genetic variants associated with UGI cancer risk [21,22]. The PRSs, gathering genetic contribution and effects of all UGI cancer-associated genetic variants, have been proven to effectively predict incident cases of ESC and GC [23,24]. Both dietary factors and genetic risk play an essential role in the development of the disease. A Gene-Diet Interaction Study from the UK Biobank showed that, compared with those in the lowest intraocular pressure (IOP) polygenic risk score (PRS) quartile who consumed no caffeine, those in the highest IOP PRS quartile who consumed ≥321 mg/day showed a 3.90-fold higher glaucoma prevalence [25]. Moreover, one current study suggested that genetic factors modified the association between diet and cardiovascular disease (CVD) [26]. However, previous studies have typically focused on the separate effects of dietary factors and genetic factors on UGI cancer risk. Few studies provided insight into the combined effect of dietary factors and genetic factors on UGI cancer risk. It is unclear whether there is a gene-diet combined effect or interaction in the risk of UGI cancer development, as well as the extent to which participants with a high genetic risk of UGI cancer can offset that risk by adhering to a healthy diet.

In this study, we conducted dietary pattern analysis based on examining the adherence to healthy diet and investigated the association of adherence to healthy diet with UGI cancer risk using UK Biobank data. We also tested the hypothesis that dietary factors and genetic factors jointly contribute to incident UGI cancer and that adopting a healthy diet can attenuate UGI cancer risk for individuals at high genetic risk.

## 2. Materials and Methods

### 2.1. Study Design and Participants

**Figure 1:**
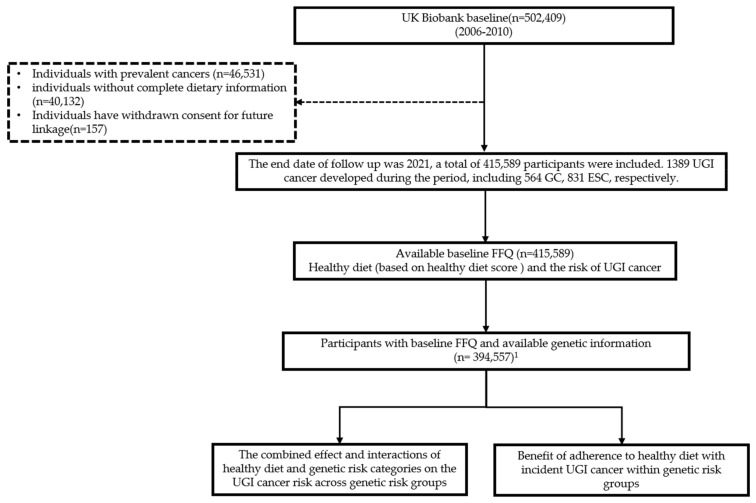
**Study design and workflow.** ^1^ For healthy diet and genetic risk on UGI cancer risk across and within genetic risk group analysis, participants without available genetic information were excluded (*n* = 21,032).

UK Biobank is a large, population-based prospective study with genetic and phenotypic data. Between 2006 and 2010, UK Biobank recruited over 500,000 participants from the general population who were aged 40–69 years. Participants were recruited at 22 assessment centers located throughout England, Wales, and Scotland [27]. Participants completed a touch-screen questionnaire, took physical measurements, and provided biological samples at assessment centers. The basic collection details are described elsewhere [28,29]. We excluded participants with prevalent cancer (*n* = 46,531), those who were missing any dietary information data (*n* = 40,132), and individuals who had withdrawn consent for future linkage (*n* = 157), leaving 415,589 participants (193,083 men and 222,506 women) included in the study. First, we examined the association between the degree of adherence to healthy diet defined by healthy diet score and UGI cancer risk. Then, we compared the combined effect and interactions of healthy diet and genetic risk categories on UGI cancer risk across genetic risk groups. Last, we compared the benefit of adherence to a healthy diet within genetic risk groups (Figure 1).

### 2.2. Exposure Measurement

#### 2.2.1. Dietary Intake Assessment

The touch-screen questionnaire, self-completed at baseline, was used to collect the frequency of consumption of the following 12 food items over the previous year with FFQ: beef, lamb, pork, processed meat, oily fish, non-oily fish, fresh fruit, dried fruit, raw vegetables, cooked vegetables, cereal, and bread. We also created new data fields based on food items: (1) Red meat intake, (2) Total fish intake, (3) Total vegetables intake, (4) Total fruit intake, (5) Whole grains intake, and (6) Refined grains intake. We summed beef, lamb and pork intake to create red meat intake. We also summed oily fish and non-oily fish intake to generate total fish intake. To calculate total vegetables and fruit consumption respectively, we aggregated cooked vegetables and salad/raw vegetable intake as total vegetables intake, and fresh fruit and dried fruit as total fruit intake. We divided grains into whole grains and refined grains according to the type of bread and cereal mainly consumed. We defined wholemeal or wholegrain bread, bran cereal, oat cereal, and muesli as whole grains; white bread, brown bread, other bread, biscuit cereal, and other cereals as refined grains. We categorized the 12 food items into 7 food groups, including red meat, processed meat, total fish, total fruit, total vegetables, whole grains and refined grains. We also defined serving size for each baseline food items. For bread and cereal, data were provided for weekly consumption, which were converted into daily consumption. Detailed serving size and coding for each food item/food group are shown in Table S1.

#### 2.2.2. Healthy Diet Score Estimation

We adopted seven dietary factors and cut-offs according to recommendations for dietary priorities on cardiometabolic health [30], that is, increasing fruit, vegetables, whole grains, and fish consumption, and decreasing red meat, processed meat, and refined grains intake. The healthy diet score was calculated using the seven dietary components: Total fruit ≥ 4 servings/day; Total vegetables ≥ 4 servings/day; Total fish ≥ 2 servings/week; Processed meat ≤ 1 serving/week; Red meat ≤ 1.5 servings/week; Whole grains ≥ 3 servings/day; Refined grains ≤ 1.5 servings/day. Each favorable dietary factor was given one point (Table S2). The score ranged from 0 to 7; we defined score 0–1 as low-quality diet, 2–4 as intermediate-quality diet, and 5–7 as high-quality diet, according to data distribution characteristics. Next, we categorized the scores into unfavorable diet (healthy diet score < 4) and favorable diet (healthy diet score ≥ 4).

### 2.3. PRS Calculation and UGI-PRS Construction

Genotyping process and single nucleotide polymorphisms (SNPs) used in the UKB research have been described elsewhere in detail [31,32]. We extracted variants with *p* < 5 × 10^−8^ and minor allele frequency (MAF) ≥0.01 from GWAS with the largest sample size in European ancestry [23,33]. For variants that were not available in the UKB genotyping data, their strong correlated SNPs (*r*^2^ > 0.8) were included in the present study. If more than one variant correlated in the same locus were reported, the SNPs with the smallest reported *p*-value were selected by using the linkage disequilibrium clumping procedure (at *r*^2^ < 0.2) in PLINK. We excluded SNPs with allele mismatches or MAF differences > 0.10, compared with those in the European population of 1000 Genomes, and palindromic SNPs (A/T, G/C) with an MAF ≥0.45. Finally, we estimated site-specific PRS based on 13 SNPs and 3 SNPs for ESC and GC, respectively (Table S3). No SNPs were shared or in high LD (*r*^2^  >  0.6) with each other in more than one site-specific PRS. Firstly, site-specific PRS was created following an additive model [34], generated by multiplying the genotype dosage of each risk allele by its respective effect size, summing all alleles together. Then, we built a UGI-PRS to assess UGI cancer risk by summing site-specific PRSs weighted by ESC and GC age-standardized incidence rate in the UK population [35]. Cancer site-specific PRS has been proven to effectively identify individuals with high risk of overall cancers and gastrointestinal cancer risk [36,37]. The UGI-PRS was divided into three levels of genetic risk: low (lowest quintile), moderate (quintiles 2–4), and high (top quintile).

### 2.4. Outcome Assessment

The outcomes in the study were first primary incident events due to UGI cancer (ESC and GC), which is identified through the national cancer registries of England, Wales, and Scotland, coded by the 10th revision of the International Classification of Diseases (ICD-10), as (C15) and (C16) for ESC and GC, respectively. After four years of baseline recruitment (2006–2010), UGI cancer risk in participants was assessed from baseline up to the UGI cancer diagnosis, death, completion of follow-up, or loss to follow-up, whichever occurred first. The time of risk was calculated according to date the participant attended the assessment center (Data Field: 53), date of cancer diagnosis (Data Field: 40005) and the end date of follow-up. The end date of follow-up was updated to September 2018 for Scotland and to June 2021 for England and Wales. For participants who developed a UGI cancer, time at risk was the interval between the date of cancer diagnosis and the date of attending assessment. For participants without UGI cancer, time at risk was calculated by the end date of follow-up minus date of attending assessment center.

### 2.5. Statistical Analysis

Cox proportional hazard models were used to investigate the associations between healthy diet and UGI cancer risk and to estimate hazards ratios (HRs) and 95% confidence intervals (CIs) with the time of follow-up used as the timeline variable. The proportional hazard assumptions were checked using Schoenfeld residuals. We determined UGI cancer risk for participants among healthy diet score categories (low-quality diet, intermediate-quality diet, and high-quality diet group). We also compared the UGI cancer risk for per two-point increase in healthy diet score. Furthermore, we investigated the combined effect and interactions of dietary and genetic factors on UGI cancer risk according to healthy diet and genetic risk categories to explore the extent to which healthy diet modified the associations between genetic susceptibility and UGI cancer risk across genetic risk groups. We examined the results for potential additive and multiplicative interaction between healthy diet and genetic risk [38]. The additive interaction was evaluated using two indexes: the relative excess risk due to the interaction (RERI) and the attributable proportion due to the interaction (AP) [39]. The 95% CIs of the RERI and AP were generated by drawing 5000 bootstrap samples from the estimation data set [40]. If there was no additive interaction, the CIs of the RERI and AP would include 0. In addition, we used RHR (ratio of HR) to evaluate the gene–diet multiplicative interactions by setting variable cross-product terms of the healthy diet with the genetic risk in the models. The 95% CIs of RHR would contain 1 if there was no multiplicative interaction. We also calculated the absolute risk as the percentage of incident UGI cancer cases occurring in each genetic risk group to compare the benefit of adherence to a healthy diet with incident UGI cancer within genetic risk groups. The absolute risk reduction was calculated according to the given groups UGI cancer incidences difference, and then the difference in five-year event rates was extrapolated among given groups. The calculation of 95% CIs for the absolute risk reduction were calculated by drawing 1000 bootstrap samples from the estimation dataset.

Two models were applied in our analyses: minimally adjusted model, adjusted for age at recruitment, sex, Townsend deprivation index, assessment center (10 regions) and ethnic background; fully adjusted model, additionally adjusted for BMI (kg/m^2^, <25, 25–29.9, ≥30), glycosylated hemoglobin (HbA1c, mmol/mol, quintiles), smoking status (never, former, current, unknown), alcohol intake frequency (never/rare, twice or less per week, at least three times per week, unknown), education (college or university degree, no degree, unknown), multimorbidity (None, ≥1, unknown), physical activity (<600 MET minutes/week, 600–3000 MET minutes/week, >3000 MET minutes/week) [41] and family cancer history (yes, no, unknown) (Table S4). We additionally adjusted the top 10 genetic principal components of ancestry in the analysis including genetic risk. Missing data were coded as missing proxies (unknown) for categorical variables, while those for continuous variables were imputed with sex-specific median values.

We performed the following sensitivity analysis to further investigate the robustness of our results: (1) excluded participants who reported that they had made a major change in their diet in the past 5 years due to illness in the past 5 years (*n* = 41,292); (2) excluded participants followed up for less than two years (*n* = 1648); (3) excluded non-white participants (*n* = 21,680).

All statistical analyses were performed with R software for version 4.2.0 (R Core Team, Auckland, CA, USA). All *p* values were two-sided and *p* < 0.05 was considered statistically significant.

## 3. Results

### 3.1. Participants and Characteristics

A total of 415,589 participants (53.54% women) had available dietary data of this study. The median follow-up period was 12.12 (interquartile range: 11.32–12.84) years for UGI cancer incidence. A total of 1389 UGI cancer developed during the period, including 564 GC and 831 ESC. The baseline characteristics of participants are shown in Table 1. For 1389 UK Biobank participants (mean [SD] age, 61.21 [6.29] years; 27.93% women) with incidents of UGI cancer had a mean (SD) BMI of 28.61 (5.19) kg/m^2^. Of all participants, the 16.99% with UGI cancer were current smokers, and 23.18% UGI cancer participants consumed alcohol at least three times per week. The 414,200 participants (mean [SD] age, 56.17 [8.09] years; 53.63% women) had a mean (SD) BMI of 27.39 (4.75) kg/m^2^ without UGI cancer. A total of 10.25% participants with UGI cancer were current smokers, and 18.29% UGI cancer participants consumed alcohol at least three times per week.

### 3.2. Healthy Diet and the Risk of UGI Cancer

The association between adherence to healthy diet and UGI cancer risk was shown in Table 2. Individuals with a high-quality diet that included high intake of fruit, vegetables, fish and whole grains and reduced amount of red meat, processed meat and refined grains had a lower risk of UGI cancer incidents compared with those in low-quality diet group, with HR of 0.76 (95% CI: 0.62–0.93, *p* = 0.009). Having a two-point increase in healthy diet score was associated with a higher UGI cancer risk, with HR of 0.90 (95% CI: 0.83–0.97, *p* = 0.006). Similar results were noted in a series of sensitivity analyses (Table S5).

### 3.3. Combined Effect and Interactions of Healthy Diet and Genetic Risk on UGI Cancer Risk

**Figure 2:**
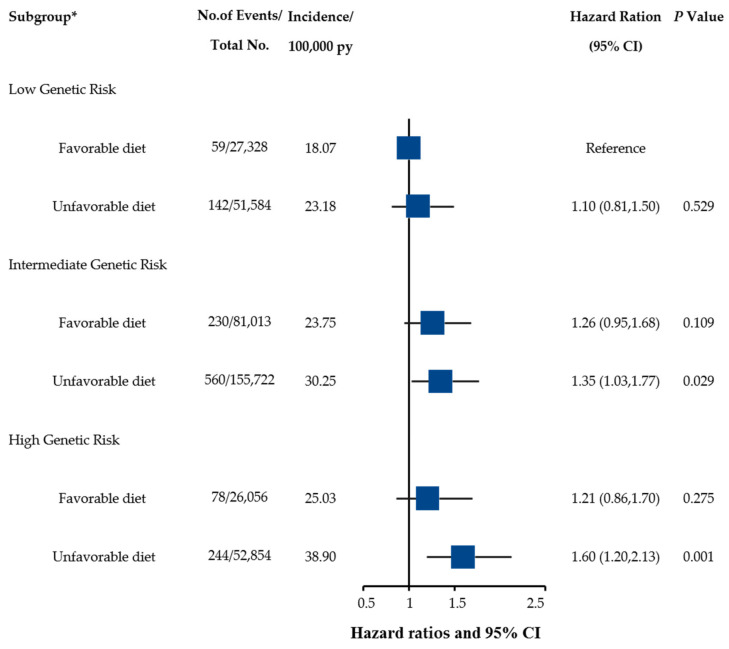
Risk of incident UGI cancer according to healthy diet and genetic risk categories in the UKB cohort. The HRs were estimated using Cox proportional hazard models with adjustment for age at recruitment, sex, assessment center (10 regions), ethnicity, Townsend deprivation index, education, BMI, glycosylated hemoglobin (HbA1c), smoking status, alcohol intake frequency, physical activity, multimorbidity, family history of cancer, and the first 10 principal components of ancestry. * For healthy diet and genetic risk on UGI cancer risk across and within genetic risk group analysis, participants without available genetic information were excluded (*n* = 21,032). Unfavorable diet (healthy diet score < 4) and Favorable diet (healthy diet score ≥ 4).

We determined that participants who had an unhealthy diet and were in a high genetic risk group had an approximately 1.60-fold risk of UGI cancer risk, with HR reaching 1.60 (95% CI: 1.20–2.13, *p* = 0.001), when compared with participants with a healthy diet and low genetic risk (Figure 2). The results of the sensitivity analysis did not change materially (Figure S1A–C). The RERI, AP, and RHR were not significant, which indicated no additive and multiplicative interactions of healthy diet and genetic risk on the risk of UGI cancer (Table 3).

### 3.4. Benefits of Adherence to a Healthy Diet with UGI Cancer Risk

In further stratification analyses with an unhealthy dietary pattern as the reference group according to genetic risk categories, we found that in the intermediate and high genetic risk groups, similar risk reduction for UGI cancer were observed in those who adhered to a healthy dietary pattern compared to those who adhered to an unhealthy dietary pattern. Among participants with an intermediate genetic risk, the absolute five-year incidence risk of UGI cancer were 0.13 for participants with an unhealthy dietary pattern versus 0.11 for those with a healthy dietary pattern. Similarly, for individuals with high genetic risk, the absolute five-year incidence risk of UGI cancer decreased from 0.16 for participants with an unhealthy dietary pattern to 0.10 for those with a healthy dietary pattern (Table 4). The results of sensitivity analyses were similarly (Table S6).

## 4. Discussion

In this large, prospective study using UK Biobank, we investigated dietary pattern analyses based on healthy diet and UGI cancer risk. We found that improving the quality of healthy diet was associated with a lower risk of UGI cancer. Across genetic risk groups, analysis further showed that individuals with high genetic risk and an unhealthy dietary pattern were at a greater risk of UGI cancer compared to those with low genetic risk and a healthy dietary pattern. Within genetic risk groups, analysis indicated that adherence to a healthy dietary pattern was consistently associated with a decreased absolute five-year incidence risk of UGI cancer in intermediate and high genetic risk groups.

Current studies suggested that dietary patterns analyses are regarded as good ways to explore diet and cancer risk. A systematic review and meta-analysis from prospective cohort studies supported an association between healthy dietary patterns and decreased risks of colon and breast cancer [42]. One study that focused on nutrition and breast cancer showed that adherence to a healthy dietary pattern might improve overall survival after diagnosis of breast cancer [43]. We performed dietary pattern analyses based on healthy diet score and the risk of UGI cancer. A systematic review and meta-analysis on dietary patterns and gastric cancer risk indicated that there is an approximately two-fold difference in GC risk between a ‘prudent/healthy’ diet, and a ‘Western/unhealthy’ diet [17]. A population-based case-control study suggested that a diet high in fruit and vegetables may decrease the risk of ESC cancer [44]. Another systematic review and meta-analysis suggested that a healthy dietary pattern was significantly associated with a decreased risk of ESC [45]. Our study also found similar results, i.e., that adherence to a healthy diet reduced the UGI cancer risk. We also compared the benefit of adherence to a healthy dietary pattern within genetic risk groups based on the calculation of absolute five-year incidence risk of UGI cancer. We found that individuals with intermediate and high genetic risk who adopted a healthy diet had a decreased risk of developing UGI cancer. For participants with high genetic risk, the absolute five-year incidence risk of UGI cancer was significantly reduced from 0.16% to 0.10% by having a healthy diet. Taken together, our findings along with previous evidence not only demonstrated the significance of adherence to healthy diet, but also provided collective support for public health interventions to promote a healthy dietary pattern for everyone, especially people with intermediate or high genetic risks, which will ultimately lead to a reduction of UGI cancer burden.

It has been estimated that ESC and GC could be prevented in 54% and 59% of patients in the UK, respectively [46]. It is important to understand the contribution of modifiable risk factors to UGI cancer and how they affect or add to the inherited genetic factors. At present, several studies have summarized the association between diet and nutrition and the UGI cancer risk; however, reported meta-analytic estimates from observational studies may not represent causality. Instead, they may result from common biases across studies, such as exposure measurement error, residual confounding, and publication bias, and thereby weaken the strength of the scientific evidence [47,48,49]. In addition, few studies have focused on the combined effect and interactions of gene–diet on the risk of UGI cancer. We systematically and comprehensively investigated the association between modifiable dietary factors with UGI cancer risk and tested the hypothesis that UGI cancer risk can be modified or reduced by adopting a healthy diet in a large prospective cohort study.

UK Biobank is a large, general population-based prospective cohort, which provides health outcomes and a wide range of potential confounders, including diet. One of the inevitable problems with large sample studies is that *p* values are more likely to be statistically different. In detail, a statistical *p* value is the distance between the data and the null hypothesis measured by an estimate of the parameter of interest. This distance is usually measured in terms of the standard deviation (standard error). The standard error shrinks as the sample size increases; in a very large sample, the standard error becomes very small, which leads to a statistically significant distance between the estimate and the null hypothesis that may be negligible. Therefore, to reduce type I errors, the null hypothesis cannot be rejected by the *p*-value alone in a large sample study. These problems can be solved by additionally reporting effect sizes and 95% confidence intervals (CI) [50]. In our study, we provided 95% CI as well as *p* values to more cautiously infer the association between healthy diet and UGI cancer.

The present study has several limitations. First, participants in the UK Biobank are of European descent; therefore, the summary statistics should be generalized to the general population with caution. Secondly, the use of self-reported recall of FFQ could introduce some level of recall bias. Third, it is generally accepted that associations between nutrients and disease should only be considered primary if the effects are independent of energy intake [51]. We were not able to adjust for total energy intake because the baseline touchscreen brief FFQ only covered some commonly consumed foods. Therefore, our findings may be biased by the differences in body size, physical activity, and metabolic efficiency resulting from energy intake. Last, covariates were evaluated only once at baseline, and changes during the follow-up or competitive risk of other illnesses may have an effect on risk estimates.

## 5. Conclusions

Our findings confirm and broaden the results from previous studies. Healthy diet was associated with a lower risk of UGI cancer. Dietary factors and genetic risk had a combined effect on risk of UGI cancer. Individuals with high genetic risk can attenuate UGI cancer risk by adopting a healthy dietary pattern.

## Figures and Tables

**Table 1:** Baseline characteristics of participants in UK Biobank ^1^.

	Participants	
	With Incident UGI	Without UGI
	Cancer (*n* = 1389)	Cancer (*n* = 414,200)
Age at baseline, y	61.21 ± 6.29	56.17 ± 8.09
Female	388 (27.93)	222,118 (53.63)
Townsend deprivation index, means ± SD	−1.04 ± 3.23	−1.40 ± 3.03
BMI, means ± SD	28.61 ± 5.19	27.39 ± 4.75
HbA1c, mmol/mol, means ± SD	38.12 ± 7.98	35.94 ± 6.47
Physical activity, MET minutes/week		
<600	259 (18.65)	63,772 (15.4)
600–3000	774 (55.72)	244,531 (59.04)
>3000	356 (25.63)	105,897 (25.57)
Ethnicity		
White	1346 (96.9)	392,733 (94.82)
Nonwhite	38 (2.74)	20,197 (4.88)
Unknown	5 (0.36)	1270 (0.31)
Education		
College or university degree	352 (25.34)	139,657 (33.72)
No degree	1023 (73.65)	271,190 (65.47)
Unknown	14 (1.01)	3353 (0.81)
Smoking status		
Never	506 (36.43)	228,680 (55.21)
Former	640 (46.08)	141,909 (34.26)
Current	236 (16.99)	42,454 (10.25)
Unknown	7 (0.5)	1157 (0.28)
Alcohol intake frequency		
Never/rare	621 (44.71)	184,431 (44.53)
Twice or less per week	445 (32.04)	153,836 (37.14)
At least three times per week	322 (23.18)	75,752 (18.29)
Unknown	1 (0.07)	181 (0.04)
Health status		
Multimorbidity, n (%)		
None	216 (15.55)	107,012 (25.84)
≥1	1172 (84.38)	306,847 (74.08)
Unknown	1 (0.07)	341 (0.08)
Family cancer history		
yes	832 (59.9)	257,969 (62.28)
no	380 (27.36)	109,695 (26.48)
Unknown	177 (12.74)	46,536 (11.24)

^1^ Values are presented as mean ± SD or *n* (%) unless otherwise indicated.

**Table 2:** Associations between healthy diet score and the risk of UGI cancer.

Healthy Diet	Total No. (Cases)	Minimally Adjusted Model ^1^		Fully Adjusted Model ^2^	
		HR (95% CI)	*p* Value	HR (95% CI)	*p* Value
Healthy diet score ^3^					
Low-quality diet (0–1)	64,171 (304)	1.00 (ref)		1.00 (ref)	
Intermediate-quality diet (2–4)	297,417 (943)	0.81 (0.71, 0.92)	0.001	0.87 (0.77, 1.00)	0.047
High-quality diet (5–7)	54,001 (142)	0.66 (0.54, 0.81)	<0.001	0.76 (0.62, 0.93)	0.009
Per two-point score increase	415,589 (1389)	0.84 (0.78, 0.91)	<0.001	0.90 (0.83, 0.97)	0.006
*p* for trend			<0.001		0.007

Definition of abbreviations: HR, hazard ratio; 95% CI: 95% confidence interval; ref, reference. 1 Minimally adjusted model: adjusted for age at recruitment, sex, assessment center (10 regions), Townsend deprivation index and ethnicity. 2 Fully adjusted model: minimally adjusted model additionally adjusted for education, BMI, glycosylated hemoglobin (HbAlc), smoking status, alcohol intake frequency, physical activity, multimorbidity and family history of cancer. 3 Healthy diet score: using available data from UK Biobank Food Frequency Questionnaire at baseline; Health diet score ranged from 0 to 7. Fruits: ≥4 servings/day; Vegetables: ≥4 servings/day; Fish: ≥2 servings/week; Processed meats: ≤1 serving/week; Unprocessed red meats: ≤1.5 servings/week; Whole grains: ≥3 servings/day; Refined grains: ≤1.5 servings/day.

**Table 3:** Interaction between diet and genetic risk ^1^.

	PRS *	
	Intermediate	High
RERI (95% CI)	−0.01 (−0.47–0.31)	0.28 (−0.23–0.67)
AP (95% CI)	−0.01 (−0.29–0.26)	0.18 (−0.13–0.45)
RHR (95% CI)	1.03 (0.73–1.45)	0.84 (0.56–1.24)

Definition of abbreviations: RERI = relative excess risk due to the interaction; AP = attributable proportion due to the interaction; RHR = ratio of hazard ratio. * Defined by PRS: low (lowest quintile), intermediate (quintiles 2–4), and high (quintile 5). ^1^ Cox proportional hazards regression is adjusted for age at recruitment, sex, assessment center (10 regions), Townsend deprivation index, ethnicity, education, BMI, glycosylated hemoglobin (HbAlc), smoking status, alcohol intake frequency, physical activity, multimorbidity, and family history of cancer.

**Table 4:** UGI cancer risk associated with healthy diet by genetic risk level in the UKB cohort ^1^.

	Low Genetic Risk		Intermediate Genetic Risk		High Genetic Risk	
Dietary Pattern	Unfavorable	Favorable	Unfavorable	Favorable	Unfavorable	Favorable
No. of cases/Person-years	142/61,2672	59/326,606	560/185,1465	230/968,645	244/627,221	78/311,605
HR (95% CI)	Ref.	0.85 (0.63–1.17)	Ref.	0.94 (0.80–1.10)	Ref.	0.78 (0.60–1.01)
*p* value		0.323		0.417		0.057
Absolute risk (%)-5 years (95% CI)	0.10 (0.07–0.12)	0.08 (0.05–0.10)	0.13 (0.12–0.15)	0.11 (0.09–0.12)	0.16 (0.13–0.19)	0.10 (0.07–0.13)
Absolute risk reduction (%)-5 years (95% CI)	Ref.	0.02 (−0.06–0.49)	Ref.	0.03 (0.01–0.05)	Ref.	0.06 (0.02–0.09)

^1^ Cox proportional hazards regression is adjusted for age at recruitment, sex, assessment center (10 regions), Townsend deprivation index, ethnicity, education, BMI, glycosylated hemoglobin (HbAlc), smoking status, alcohol intake frequency, physical activity, multimorbidity and family history of cancer. Unfavorable dietary pattern (healthy diet score < 4) and Favorable dietary pattern (healthy diet score ≥ 4).

## Data Availability

Since the UK Biobank has proprietary rights to the data, data used for this analysis and the code book included in the manuscript are not publicly available. External researchers can apply to use the UK Biobank data set by registering and applying at the website: http://www.ukbiobank.ac.uk/register-apply (accessed on 11 November 2022).

## Supplementary Materials

The following supporting information can be downloaded at: https://www.mdpi.com/article/10.3390/nu15061344/s1, Figure S1A: Risk of incident UGI cancer according to healthy diet and genetic risk categories in the UKB cohort after excluding participants who report changing their diet in the last 5 years due to illness; Figure S1B: Risk of incident UGI cancer according to genetic and healthy diet and genetic risk categories in the UKB cohort after excluding participants who report less than 2 years of follow-up; Figure S1C: Risk of incident UGI cancer according to healthy diet and genetic risk categories in the UKB cohort after excluding non-white participants; Table S1: Serving size and coding of intake for each touchscreen food items/food groups; Table S2: Healthy diet score factors definition; Table S3: Single nucleotide polymorphisms utilized to build the polygenic risk scores for UGI cancer; Table S4: Definition of covariates; Table S5: Associations between healthy diet and the risk of UGI cancer after excluding participants who report changing their diet due to in the last 5 years due to illness or after excluding participants who report less than 2 years of follow-up or after excluding non-white participants; Table S6: UGI cancer risk associated with healthy diet by genetic risk level after excluding participants who report changing their diet in the last 5 years due to illness or after excluding participants who report less than 2 years of follow-up or after excluding non-white participants.
