# Supplementary material for: Healthy Diet, Polygenic Risk Score, and Upper Gastrointestinal Cancer Risk: A Prospective Study from UK Biobank
Source: Nutrients. 2023 Mar 10;15(6):1344. doi: 10.3390/nu15061344 (PMC10054787; doi:10.3390/nu15061344)
Supplement: Supplementary file 1 [file nutrients-15-01344-s001.zip › nutrients-2258523-supplementary.pdf]

Supplementary Material

Contents

Figure S1A. Risk of incident UGI cancer according to priori dietary pattern and genetic risk categories in the UKB cohort after excluding participants who report changing their diet in the last 5 years due to illness

Figure S1B. Risk of incident UGI cancer according to priori dietary pattern and genetic risk categories in the UKB cohort after excluding participants who report less than 2 years of follow-up

Figure S1C. Risk of incident UGI cancer according to priori dietary pattern and genetic risk categories in the UKB cohort after excluding non-white participants

Table S1. Serving size and coding of intake for each touchscreen food items/food groups

Table S2. Healthy diet score factors definition

Table S3. Single nucleotide polymorphisms utilized to build the polygenic risk scores for UGI cancer

Table S4. Definition of covariates

Table S5. Associations between healthy diet score and the risk of UGI cancer after excluding participants who report changing their diet due to in the last 5 years due to illness or after excluding participants who report less than 2 years of follow-up or after excluding non-white participants

Table S6. UGI cancer risk associated with priori dietary pattern by genetic risk level after excluding participants who report changing their diet in the last 5 years due to illness or after excluding participants who report less than 2 years of follow-up or after excluding non-white participants

1.1 Figures

Subgroup	No.of Events/ Total No.	Incidence/ 100,000 py	Hazard Ration (95% CI)	*P* Value
Low Genetic Risk				
Favorable diet	48/24,565	16.32	Reference	
Unfavorable diet	120/46,458	21.70	1.15 (0.82,1.61)	0.419
Intermediate Genetic Risk				
Favorable diet	188/72,899	21.53	1.28 (0.93,1.76)	0.131
Unfavorable diet	478/140,862	28.48	1.42 (1.05,1.92)	0.021
High Genetic Risk				
Favorable diet	66/23,407	23.53	1.27 (0.87,1.84)	0.209
Unfavorable diet	210/47,627	37.04	1.70 (1.24,2.33)	0.001

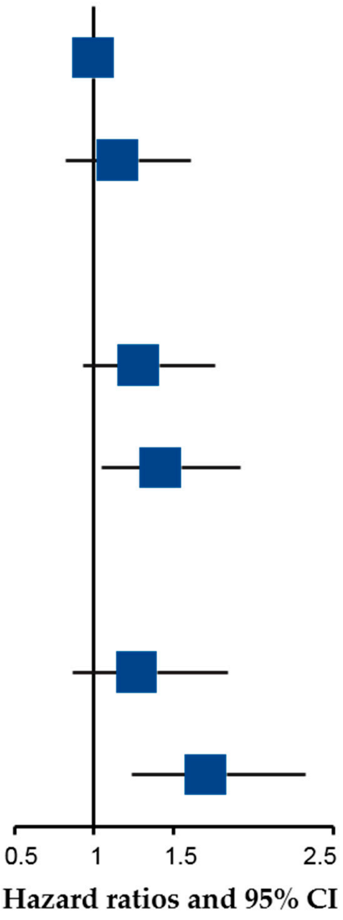

Figure S1A. Risk of incident UGI cancer according to healthy diet and genetic risk categories in the UKB cohort after excluding participants who report changing their diet in the last 5 years due to illness. The HRs were estimated using Cox proportional hazard models with adjustment for age at recruitment, sex, geographical region (10 regions), ethnicity, Townsend deprivation index, education, BMI, glycosylated hemoglobin (HbA1c) smoking status, alcohol intake frequency, physical activity, multimorbidity, family history of cancer and the first 10 principal components of ancestry.

Subgroup	No.of Events/ Total No.	Incidence/ 100,000 py	Hazard Ration (95% CI)	*P* Value
Low Genetic Risk				
Favorable diet	54/27,258	16.54	Reference	
Unfavorable diet	128/51,377	20.90	1.10 (0.80,1.51)	0.569
Intermediate Genetic Risk				
Favorable diet	187/80,736	19.31	1.13 (0.84,1.53)	0.423
Unfavorable diet	488/155,043	26.37	1.31 (0.98,1.74)	0.064
High Genetic Risk				
Favorable diet	69/25,969	22.15	1.18 (0.83,1.69)	0.358
Unfavorable diet	220/52,605	35.09	1.60 (1.18,2.17)	0.002

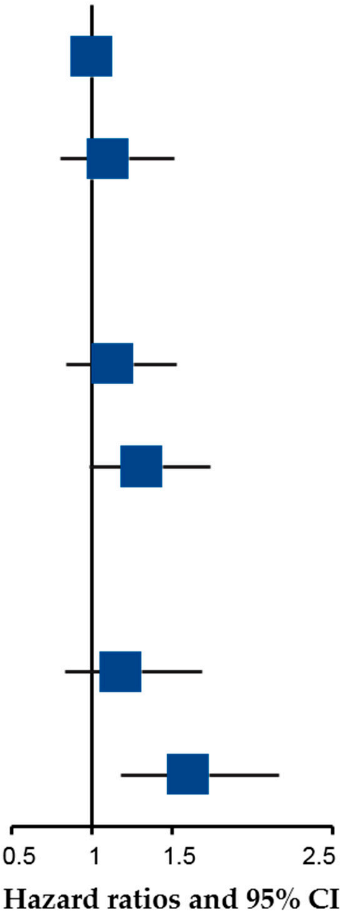

Figure S1B. Risk of incident UGI cancer according to healthy diet and genetic risk categories in the UKB cohort after excluding participants who report less than 2 years of follow-up. The HRs were estimated using Cox proportional hazard models with adjustment for age at recruitment, sex, geographical region (10 regions), ethnicity, Townsend deprivation index, education, BMI, glycosylated hemoglobin (HbA1c) smoking status, alcohol intake frequency, physical activity, multimorbidity, family history of cancer and the first 10 principal components of ancestry.

Subgroup	No.of Events/ Total No.	Incidence/ 100,000 py	Hazard Ration (95% CI)	*P* Value
Low Genetic Risk				
Favorable diet	56/24,609	19.00	Reference	
Unfavorable diet	134/47,435	23.77	1.07 (0.78,1.46)	0.670
Intermediate Genetic Risk				
Favorable diet	222/76,595	24.22	1.24 (0.92,1.66)	0.156
Unfavorable diet	545/149,311	30.70	1.32 (1.00,1.74)	0.052
High Genetic Risk				
Favorable diet	77/24941	25.80	1.20 (0.85,1.69)	0.307
Unfavorable diet	238/51161	39.19	1.54 (1.15,2.07)	0.004

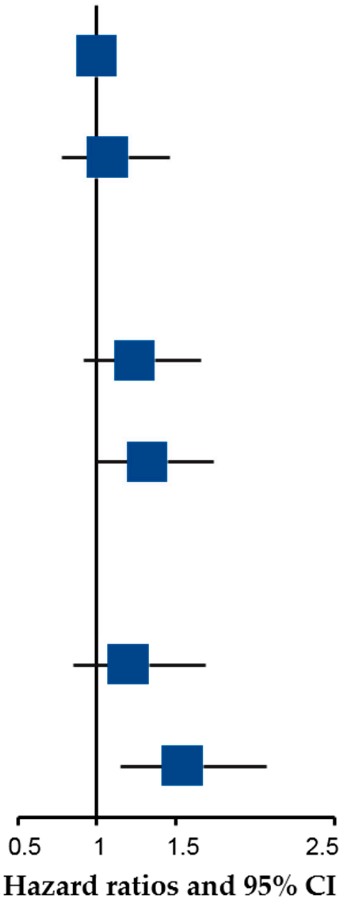

Figure S1C. Risk of incident UGI cancer according to healthy diet and genetic risk categories in the UKB cohort after excluding non-white participants. The HRs were estimated using Cox proportional hazard models with adjustment for age at recruitment, sex, geographical region (10 regions), ethnicity, Townsend deprivation index, education, BMI, glycosylated hemoglobin (HbA1c) smoking status, alcohol intake frequency, physical activity, multimorbidity, family history of cancer and the first 10 principal components of ancestry.

1.2 Tables

Table S1. Serving size and coding of intake for each touchscreen food items/food groups

Food groups	Food items	Field IDs	Amount per serving	Coding
Red meat	Beef	1369 (beef/week)	Once/week	'Never' = 0, 'Less than once a week' = 0.5, 'Once a week' = 1, '2-4 times a week' = 3, '5-6 times a week' = 5.5, 'Once or more daily' = 7
	Lamb/mutton	1379 (lamb or mutton/week)	Once/week	'Never' = 0, 'Less than once a week' = 0.5, 'Once a week' = 1, '2-4 times a week' = 3, '5-6 times a week' = 5.5, 'Once or more daily' = 7
	Pork	1389 (pork/week)	Once/week	'Never' = 0, 'Less than once a week' = 0.5, 'Once a week' = 1, '2-4 times a week' = 3, '5-6 times a week' = 5.5, 'Once or more daily' = 7
Processed meat	Processed meat	1349 (processed meat/week or daily)	Once/week	'Never' = 0, 'Less than once a week' = 0.5, 'Once a week' = 1, '2-4 times a week' = 3, '5-6 times a week' = 5.5, 'Once or more daily' = 7
Total fish	Oily fish	1329 (oily fish/week)	Once/week	'Never' = 0, 'Less than once a week' = 0.5, 'Once a week' = 1, '2-4 times a week' = 3, '5-6 times a week' = 5.5, 'Once or more daily' = 7
	Non-oily fish	1339 (non-oily fish/week)	Once/week	'Never' = 0, 'Less than once a week' = 0.5, 'Once a week' = 1, '2-4 times a week' = 3, '5-6 times a week' = 5.5, 'Once or more daily' = 7
Total fruit	Fresh fruit	1309 (pieces fresh fruit/day)	1 piece	'Less than one' = 0.5
	Dried fruit	1319 (pieces dried fruit/day)	2 pieces	'Less than one' = 0.5

Total vegetables	Cooked vegetables	1289 (tablespoons cooked vegetables/day)	2 heaped tablespoons	'Less than one' = 0.5
	Salad/raw vegetables	1299 (tablespoons salad/raw vegetables/day)	2 heaped tablespoons	'Less than one' = 0.5
Whole grains	Wholemeal/whole grain bread	1438-1 slice/week	1 slice/day	'Less than one' = 0.5
	Bran/oat/muesli cereal	1458-1 bowl/week	1 bowl/day	'Less than one' = 0.5
Refined grains	White, brown, other bread	1438-1 slice/week	1 slice/day	'Less than one' = 0.5
	Biscuit, other cereals	1458-1 bowl/week	1 bowl/day	'Less than one' = 0.5

Table S2. Healthy diet score definition

Healthy diet score factors	Intake goal	Field IDs	Amount per serving
Total fruits	≥ 4 servings/day	1309 (pieces fresh fruit/day)<br>1319 (pieces dried fruit/day)	1309 – 1 piece<br>1319 – 2 pieces
Total vegetables	≥ 4 servings/day	1289 (tablespoons cooked vegetables/day)<br>1299 (salad/raw vegetables/day)	2 heaped tablespoons
Total fish	≥2 servings/week	1329 (oily fish/week)<br>1339 (non-oily fish/week)	Once/week
Processed meats	≤ 1 servings/week	1349 (processed meat/week or daily)	Once/week
Red meat	≤ 1.5 servings/week	1369 (beef/week)<br>1379 (lamb or mutton/week)<br>1389 (pork/week)	Once/week
Whole grains	≥ 3 servings/day	1438, 1448 (wholemeal/wholegrain bread slices/week)<br>1458, 1468 (bran/oat/muesli cereal bowls/week)	1438/1448 – 1 slice/day<br>1458/1468 – 1 bowl/day
Refined grains	≤ 1.5 servings/day	1438, 1448 (white, brown, other bread slices/week)<br>1458, 1468 (biscuit, other cereals/week)	1438/1448 – 1 slice/day<br>1458/1468 – 1 bowl/day

Table S3. Single nucleotide polymorphisms utilized to build the polygenic risk scores for UGI cancer

Index	ICD10-code	Cancer site	ASR[a]	No. of SNPs included	SNP rsID[b]	Chr.	Position (GRCh37)	Risk Allele	Other Allele	Weight	Study (PMID)	Comment
1	C15	Oesophagus	14.7	13	rs3072	2	20878406	C	T	0.131028262	30243622	-
2	C15	Oesophagus	14.7	13	rs7255	2	20878820	T	C	0.157003749	30243622	Removed after LD clumping
3	C15	Oesophagus	14.7	13	rs13397172	2	200045039	C	T	0.122217633	30243622	-
4	C15	Oesophagus	14.7	13	rs2687202	3	70929983	T	C	0.122217633	30243622	-
5	C15	Oesophagus	14.7	13	rs9823696	3	183783353	A	G	0.157003749	30243622	-
6	C15	Oesophagus	14.7	13	rs62423175	6	62195368	A	G	0.207014169	30243622	-
7	C15	Oesophagus	14.7	13	rs2188554	7	117040117	A	G	0.207014169	30243622	Removed after LD clumping
8	C15	Oesophagus	14.7	13	rs17451754	7	117256712	G	A	0.223143551	30243622	-
9	C15	Oesophagus	14.7	13	rs17749155	8	10068073	A	G	0.131028262	30243622	-
10	C15	Oesophagus	14.7	13	rs10108511	8	11435516	T	C	0.113328685	30243622	-
11	C15	Oesophagus	14.7	13	rs11789015	9	96716028	A	G	0.182321557	30243622	-
12	C15	Oesophagus	14.7	13	rs7852462	9	100310501	C	T	0.076961041	30243622	-
13	C15	Oesophagus	14.7	13	rs1247942	12	114673723	G	C	0.104360015	30243622	Unspecified allele
14	C15	Oesophagus	14.7	13	rs66725070	15	58267416	GACAT	-	0.139761942	30243622	Unavailable in UK Biobank
15	C15	Oesophagus	14.7	13	rs2464469	15	58362025	G	A	0.104360015	30243622	-
16	C15	Oesophagus	14.7	13	rs1979654	16	86396835	G	C	0.104360015	30243622	Unspecified allele
17	C15	Oesophagus	14.7	13	rs10419226	19	18803172	T	G	0.165514438	30243622	-
18	C15	Oesophagus	14.7	13	rs10423674	19	18817903	C	A	0.173953307	30243622	-
19	C16	Stomach	9.9	3	rs140081212	1	153451599	G	A	0.235722334	26098866	Unavailable in UK Biobank
20	C16	Stomach	9.9	3	rs760077	1	153445406	T	A	0.235722334	26098866	-
21	C16	Stomach	9.9	3	rs6676150	1	153390461	G	C	0.235722334	26098866	Removed after LD clumping
22	C16	Stomach	9.9	3	rs2920295	8	143764937	G	A	0.19062036	26098866	-
23	C16	Stomach	9.9	3	rs10036575	5	40685795	T	C	0.210721031	26098866	-

[a]The age-standardized incidence rates (ASR, per 100,000 person-years) of each cancer in UK was assessed from the Office for National Statistics (https://www.ons.gov.uk).

[b]Single Nucleotide Polymorphisms (SNPs) were selected from previously published GWASs with the largest sample size for each cancer.

Table S4. Definition of covariates

**Covariates**	**Definition**	**UK Biobank Field code**
Age at recruitment	UK Biobank Touchscreen questionnaire at baseline; age at recruitment	21003
Sex	UK Biobank Touchscreen questionnaire at baseline; Male, female.	31
Townsend Deprivation Index	UK Biobank Touchscreen questionnaire at baseline; A score derived from national census data about car ownership, household overcrowding, owner occupation, and unemployment aggregated for postcodes of residence	189
Geographical region	UK Biobank Touchscreen questionnaire at baseline; East Midlands, London, North Eastern England, North West England, Scotland, South East England, South west England, Wales, West Midlands England, Yorkshire and the Humber	54
Ethnity	UK Biobank Touchscreen questionnaire at baseline; white, nonwhite and unknown	21000
Education	UK Biobank Touchscreen questionnaire at baseline; College or University degree, No degree (Other professional qualifications eg nursing, A levels/AS levels or equivalent, O levels/GCSEs or equivalent, CSEs or equivalent, NVQ or HND or HNC or equivalent, None of the above) and unknown	6138
BMI	UK Biobank Touchscreen questionnaire at baseline; <25 kg/m2, 25–29.9 kg/m2, ⩾30kg/m2	21001
HbA1c	UK Biobank Touchscreen questionnaire at baseline; quintiles, lowest fifth<=32.3 mmol/mol, 2 fifth(32,3-34.6 mmol/mol), 3 fifth(34.6-35.9 mmol/mol), 4 fifth(35.9-38.4 mmol/mol), highest fifth >38.4 mmol/mol)	30750
Smoking status	UK Biobank Touchscreen questionnaire at baseline; never, former, current, unknown	20116
Alcohol consumption	UK Biobank Touchscreen questionnaire at baseline; never/rare, twice or less per week, at least three times per week, unknown	20117
Physical activity	UK Biobank Touchscreen questionnaire at baseline; summed MET minutes per week for walking, MET minutes per week for moderate activity and MET minutes per week for vigorous activity. (low:<600 MET minutes/week, intermediate: 600-3000 MET minutes/week, high: >3000 MET minutes/week)	22037, 22038, 22039
Number of non-cancer illnesses	UK Biobank Touchscreen questionnaire at baseline; None, ⩾1, unknown	135
Family history of cancer	UK Biobank Touchscreen questionnaire at baseline; No: None, Yes: at least one family member had ever been diagnosed with a cancer	20107; 20110; 20111

Table S5. Associations between healthy diet and the risk of UGI cancer after excluding participants who report changing their diet in the last 5 years due to illness or after excluding participants who report less than 2 years of follow-up or after excluding non-white participants.[1]

**Healthy diet**	**after excluding participants reported changing their diet due to illness**			**after excluding participants who report less than 2 years of follow-up**			**after excluding non-white participants**		
	**Total No. (cases)***	**HR (95%CI)**	***P* value**	**Total No. (cases)***	**HR (95%CI)**	***P* value**	**Total No. (cases)***	**HR (95%CI)**	***P* value**
Healthy diet score[2]									
Low diet quality (0-1)	57833 (255)	1.00 (ref)		63829 (278)	1.00 (ref)		62064 (301)	1.00 (ref)	
Intermediate diet quality (2-4)	268354 (811)	0.90 (0.78,1.04)	0.152	296284 (818)	0.82 (0.71,0.94)	0.005	281515 (909)	0.87 (0.76,0.99)	0.035
High diet quality (5-7)	48370 (113)	0.73 (0.58,0.91)	0.006	53841 (119)	0.69 (0.55,0.85)	0.001	50500 (136)	0.76 (0.62,0.93)	0.009
Per 2 score increase	374557 (1179)	0.88 (0.81,0.95)	0.002	413954 (1215)	0.86 (0.79,0.94)	<0.001	394079 (1346)	0.90 (0.83,0.97)	0.007
P for trend			0.007			<0.001			0.006

Definition of abbreviations: HR, hazard ratio; 95%CI, 95% confidence interval; ref, reference.

[1] HR adjusted for age at recruitment, sex, geographical region (10 regions), Townsend deprivation index, ethnicity, education, BMI, Hb1Ac, smoking status, alcohol intake frequency, physical activity, multimorbidity and family history of cancer.

[2] Healthy diet score: using available data from UK Biobank Food Frequency Questionnaire at baseline; Health diet score ranged from 0 to 7. Fruits: ⩾ 4 servings/day; Vegetables: ⩾ 4 servings/day; Fish: ⩾2 servings/week; Processed meats: ⩽ 1 serving/week; Unprocessed red meats: ⩽ 1.5 servings/week; Whole grains: ⩾ 3 servings/day; Refined grains: ⩽1.5 servings/day.

Table S6. UGI cancer risk associated with healthy diet by genetic risk level after excluding participants who report changing their diet in the last 5 years due to illness or after excluding participants who report less than 2 years of follow-up or after excluding non-white participants[1]

Healthy diet	Low genetic risk		Intermediate genetic risk		High genetic risk	
	Unfavorable	Favorable	Unfavorable	Favorable	Unfavorable	Favorable
**after excluding participants who report changing their diet in the last 5 years due to illness**						
No. of cases/Person-years	120/552983	48/294044	478/1678485	188/873048	210/567001	66/280449
HR (95% CI)	Ref.	0.81 (0.58-1.15)	Ref.	0.91 (0.76-1.08)	Ref.	0.76 (0.57-1.01)
P value		0.240		0.272		0.058
Absolute risk (%)-5years (95%CI)	0.10 (0.07-0.12)	0.07 (0.05-0.10)	0.13 (0.11-0.15)	0.10 (0.08-0.11)	0.15 (0.12-0.18)	0.09 (0.07-0.12)
Absolute risk reduction (%)-5years (95% CI)	Ref.	0.02 (0.00, 0.05)	Ref.	0.03 (0.01, 0.05)	Ref.	0.05 (0.02, 0.08)
**after excluding participants who report less than 2 years of follow-up**						
No. of cases/Person-years	128/ 612424	54/326519	488/ 1850682	187/968322	220/626948	69/311504
HR (95% CI)	Ref.	0.86 (0.62-1.19)	Ref.	0.87 (0.73-1.03)	Ref.	0.76 (0.58-1.00)
P value		0.364		0.104		0.049
Absolute risk (%)-5years (95%CI)	0.07 (0.05-0.09)	0.06 (0.04-0.08)	0.09 (0.07-0.10)	0.06 (0.05-0.07)	0.11 (0.08-0.14)	0.07 (0.05-0.09)
Absolute risk reduction (%)-5years (95% CI)	Ref.	0.01 (-0.01-0.04)	Ref.	0.02 (0.01-0.03)	Ref.	0.04 (0.02-0.06)
**after excluding non-white participants**						
No. of cases/Person-years	134/563698	56/294667	545/1775543	222/916570	238/607289	77/298501
HR (95% CI)	Ref.	0.90 (0.65-1.23)	Ref.	0.94 (0.80-1.10)	Ref.	0.79 (0.61-1.03)
P value		0.500		0.441		0.084
Absolute risk (%)-5years (95%CI)	0.10 (0.08-0.13)	0.08 (0.06-0.11)	0.14 (0.12-0.16)	0.11 (0.09-0.13)	0.16 (0.12-0.19)	0.10 (0.08-0.13)
Absolute risk reduction (%)-5years (95% CI)	Ref.	0.02 (-0.01-0.05)	Ref.	0.03 (0.01-0.05)	Ref.	0.05 (0.02-0.09)

[1] HR adjusted for age at recruitment, sex, geographical region (10 regions), Townsend deprivation index, ethnicity, education, BMI, glycosylated hemoglobin (HbAlc), smoking status, alcohol intake frequency, physical activity, multimorbidity and family history of cancer.
